# N-terminal pro-brain natriuretic peptide levels in patients with anomalous left coronary artery from pulmonary artery

**DOI:** 10.1186/s13019-020-1043-3

**Published:** 2020-01-13

**Authors:** Ling Yunfei, Fan Qiang, Wang Yue, Qian Yongjun

**Affiliations:** Department of Cardiovascular Surgery, West China Hospital, Sichuan University, Guoxuexiang 37th, 610041 Chengdu, Sichuan People’s Republic of China

**Keywords:** N-terminal pro-brain natriuretic peptide, Anomalous left coronary artery from the pulmonary artery, Mitral regurgitation

## Abstract

**Introduction:**

N-terminal pro-brain natriuretic peptide (NT-pro-BNP) is used as an important biomarker for heart failure in children and adults. Previous researches have shown the value of NT-pro-BNP in various congenital heart defects (CHD). However, the level of NT-pro-BNP in patients with anomalous left coronary artery from the pulmonary artery (ALCAPA) has not been determined.

**Materials and methods:**

Plasma NT-pro-BNP was measured in 23 patients diagnosed with ALCAPA before operation. Echocardiogram was also recorded for each patient.

**Results:**

Patients with NT-pro-BNP above 300 pg/mL showed a statistically significant decrease in LVEF (*p* < 0.0001) and in age (p < 0.0001) compared to patients with NT-pro-BNP below 300 pg/mL. Age (*r* = 0.399, *p* = 0.012) and LVEF (*r* = 0.403, *p* = 0.011) showed a statistically significant correlation with NT-pro-BNP in linear regression when NT-pro-BNP more than 300 pg/mL. A negative correlation was shown between NT-pro-BNP and LVEF (*r* = 0.570, *p* < 0.0001) in all the patients. No significant correlation was observed between mitral regurgitation (MR) grade and NT-pro-BNP in a Spearman correlation test (*r* = 0.383; *P* = 0.071).

**Conclusions:**

In patients with ALCAPA, NT-pro-BNP levels showed a negative correlation with age and LVEF when NT-pro-BNP above 300 pg/mL and no correlation with age and LVEF when NT-pro-BNP under 300 pg/ml. Further studies are needed to determine whether there is a correlation between MR grade and NT-pro-BNP levels.

## Introduction

The main site of brain natriuretic peptide (BNP) synthesis and release is the cardiac ventricle, which releases BNP in response to stretch due to increased blood volume or pressure. Previous work has established the role of NT-pro-BNP in the diagnosis and treatment of heart failure (HF) [1, 2]. NT-pro-BNP has also been indicated as a strong independent prognostic factor in patients with various congenital heart disease*s (CHD)* [3–6]. However, the presentation of natriuretic peptides in ALCAPA has rarely been reported.

ALCAPA is a rare but potentially fatal CHD which is associated with adult sudden death and early infant mortality. Currently the most effective treatment is surgical coronary artery rebuilding while the prognosis is poor if left untreated [7]. ALCAPA is conventionally divided into two types, which present in infants or adults. In infants, little or no collateral coronary circulation develops and there is early onset of symptoms when pulmonary artery (PA) pressure falls after closure of the ductus arteriosus [8, 9]. Without surgical intervention, most patients die within several months due to LV dysfunction. Survival of adult patients relies on a dominant right coronary artery (RCA) with extensive coronary collaterals and a restrictive opening between the PA and ALCAPA [7, 10–13]. With ongoing subclinical myocardial ischemia, these patients may be asymptomatic until adulthood. At a mean age of 35 years, there is an estimated 80% incidence of sudden death in the adults [11–13]. Arrhythmias and ventricle dysfunction triggered by inadequate perfusion are the main causes of complications in patients with ALCAPA [13]. Mortality and morbidity of ALCAPA patients were associated with LVEF and MR, finally associated with heart function. The role of NT-pro-BNP in the diagnosis and treatment of heart failure is distinct. However the relationship between NT-pro-BNP and LVEF, the grade of MR in patients with ALCAPA remained unknown.

The aim of our study was to identify the difference in NT-pro-BNP levels between the two types of ALCAPA and if there was any correlation between elevated NT-pro-BNP and LVEF or MR grade.

## Materials and methods

Twenty-three patients diagnosed with ALCAPA, including 12 male and 11 female patients, were enrolled in the study: 15 of them were younger than 3-years-old. Cardiac function was assessed by calculating the LVEF and the grade of MR in all the patients. Blood samples were obtained from each patient on admission and plasma NT-pro-BNP was analyzed using the Elecsys 2010 electrochemiluminescence immunoassay (Roche Diagnostics, Mannheim, Germany). Statistical analyses was performed using SPSS 25.0 or GraphPad Prism 7, **Continuous variables were analyzed with a Wilcoxon rank-sum test, as assumptions of normality could not always be satisfied. Dichotomous outcomes were analyzed using Fisher’s exact test** and a value of *P* < 0.05 was considered statistically significant.

## Results

Changes in NT-pro-BNP level correlates with LVEF and age but not with MR grade. Previous studies have established the use of NT-pro-BNP levels in quantitative diagnosis of HF, specifically values < 300 pg/mL may be used to “rule out” HF in young adults [14]. In this study, NT-pro-BNP levels were used to divide the demographic, echocardiographic, and analytical data of the patients (Table 1) into two groups with plasma NT-pro-BNP values less than or more than 300 pg/mL. The mean ± standard deviation of plasma NT-pro-BNP values was 85.14 ± 49.02 pg/mL in the < 300 pg/mL group and 12,398.25 ± 8486.73 pg/mL in the > 300 pg/mL group. In a comparison of these two groups, age (*p* < 0.0001) and LVEF (p < 0.0001) showed statistical significance with the cutoff point of 300 pg/mL.

**Table 1:** Clinical, echocardiographic, and analytical data of patients with ALCAPA

Variable	NT-pro-BNP (pg/mL)		p
	< 300	> 300	
Total of patients, N	8	15	
Age, year	25.4 ± 18.23	1.2 ± 1.02	< 0.0001
Male, N	5	7	1.000
LVEF, %	61.13 ± 9.58	37.53 ± 12.34	< 0.0001
Mitral regurgitation (grade)	1.00 ± 0.71	1.87 ± 0.81	0.0232

*NT-pro-BNP* N-terminal pro-brain natriuretic peptide, *N* Number of patients, *LVEF* Left ventricular ejection fraction

**Fig. 1:**
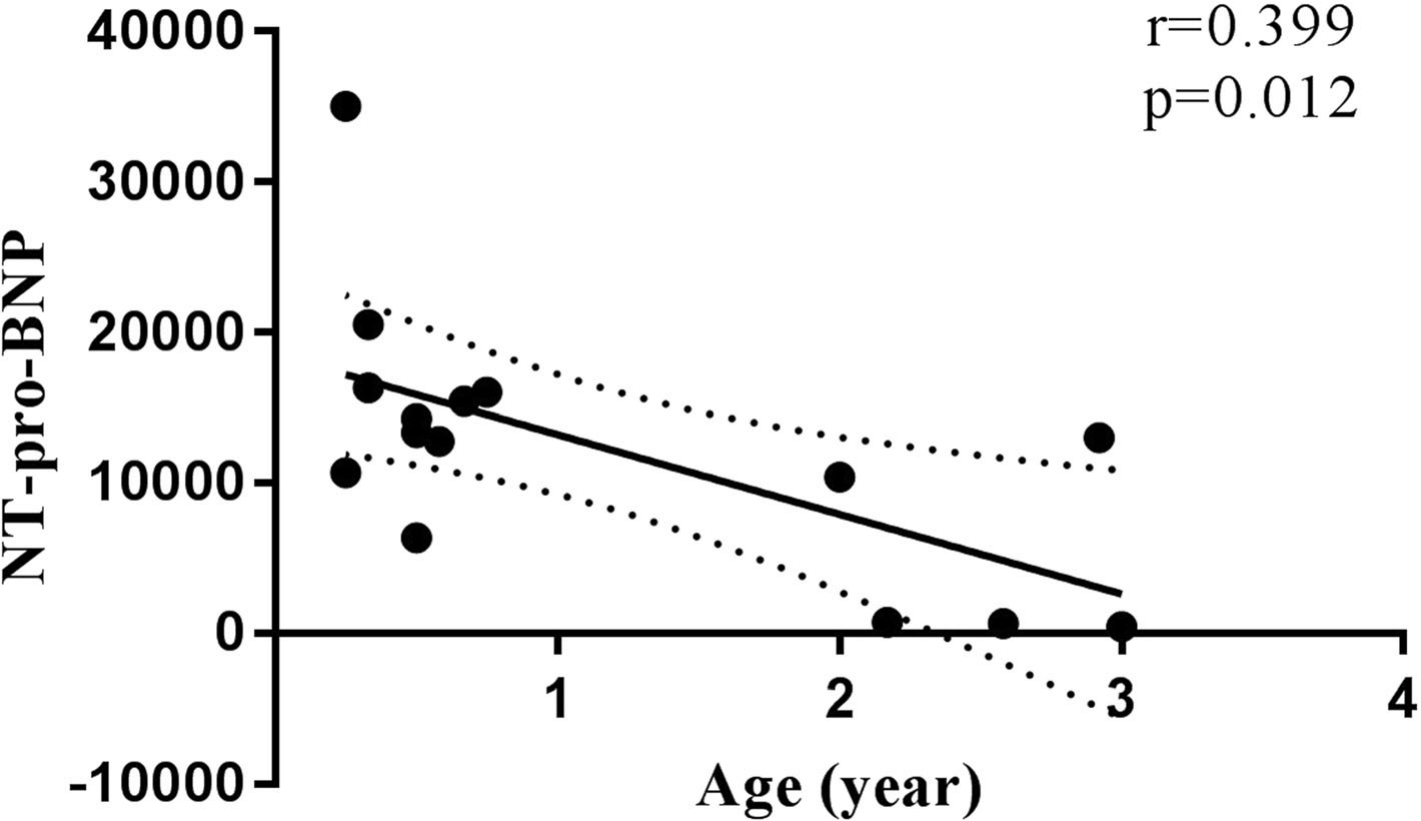
Linear regression showed a negative correlation between age and NT-pro-BNP levels (*r* = 0.399, *p* = 0.012) when NT-pro-BNP levels were above 300 pg/mL

The correlation between NT-pro-BNP and age in the two groups was analyzed by linear regression. A statistically significant negative correlation was observed between age (*r* = 0.399, *p* = 0.012) and NT-pro-BNP when NT-pro-BNP levels were above 300 pg/mL (Fig. 1). However, when NT-pro-BNP levels were below 300 pg/mL, there was no statistically significant correlation between age and NT-pro-BNP (*r* = 0.060, *p* = 0.560).

**Fig. 2:**
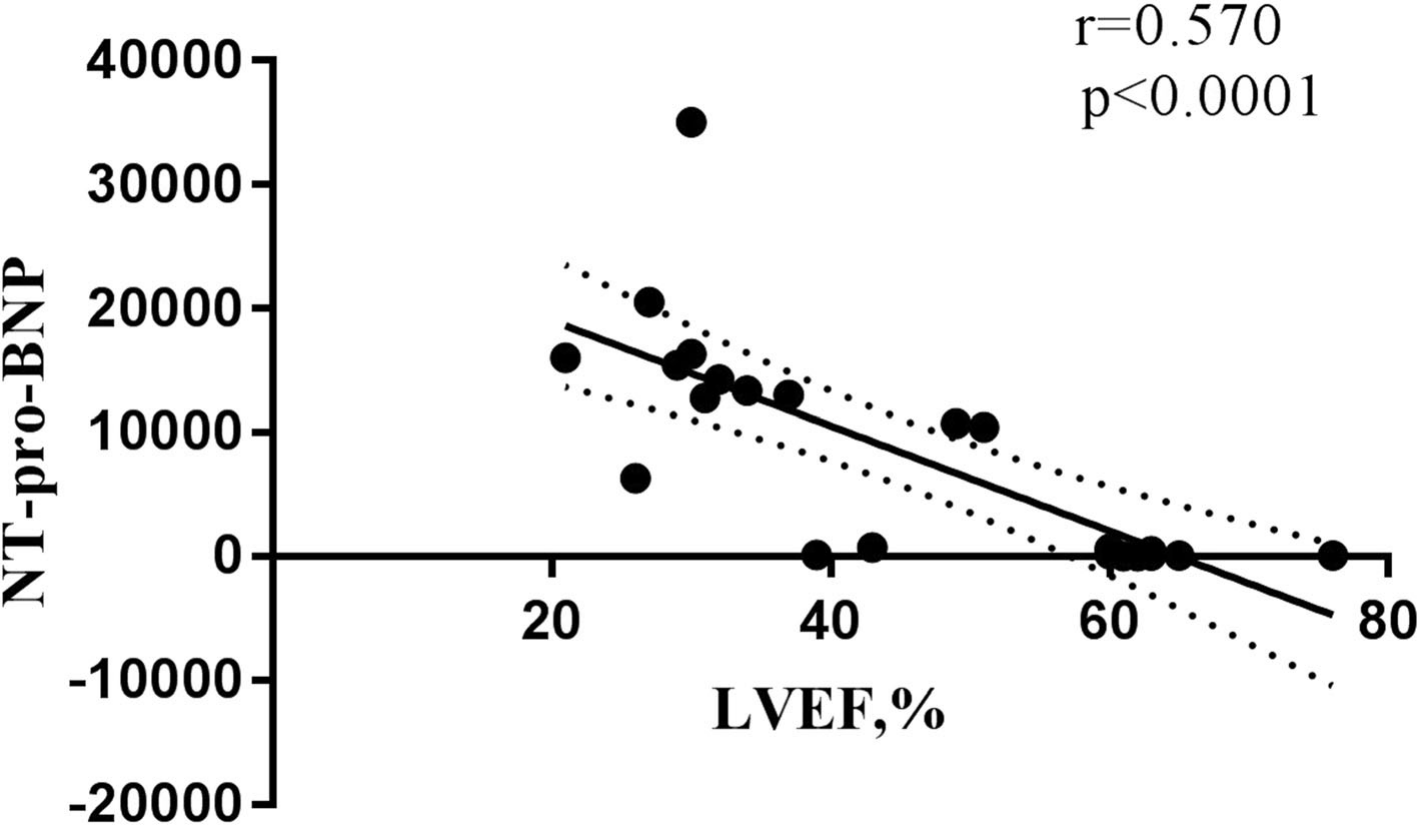
Linear regression showed a negative correlation between LVEF and NT-pro-BNP levels (*r* = 0.570, *p* < 0.0001) in patients with ALCAPA

**Fig. 3:**
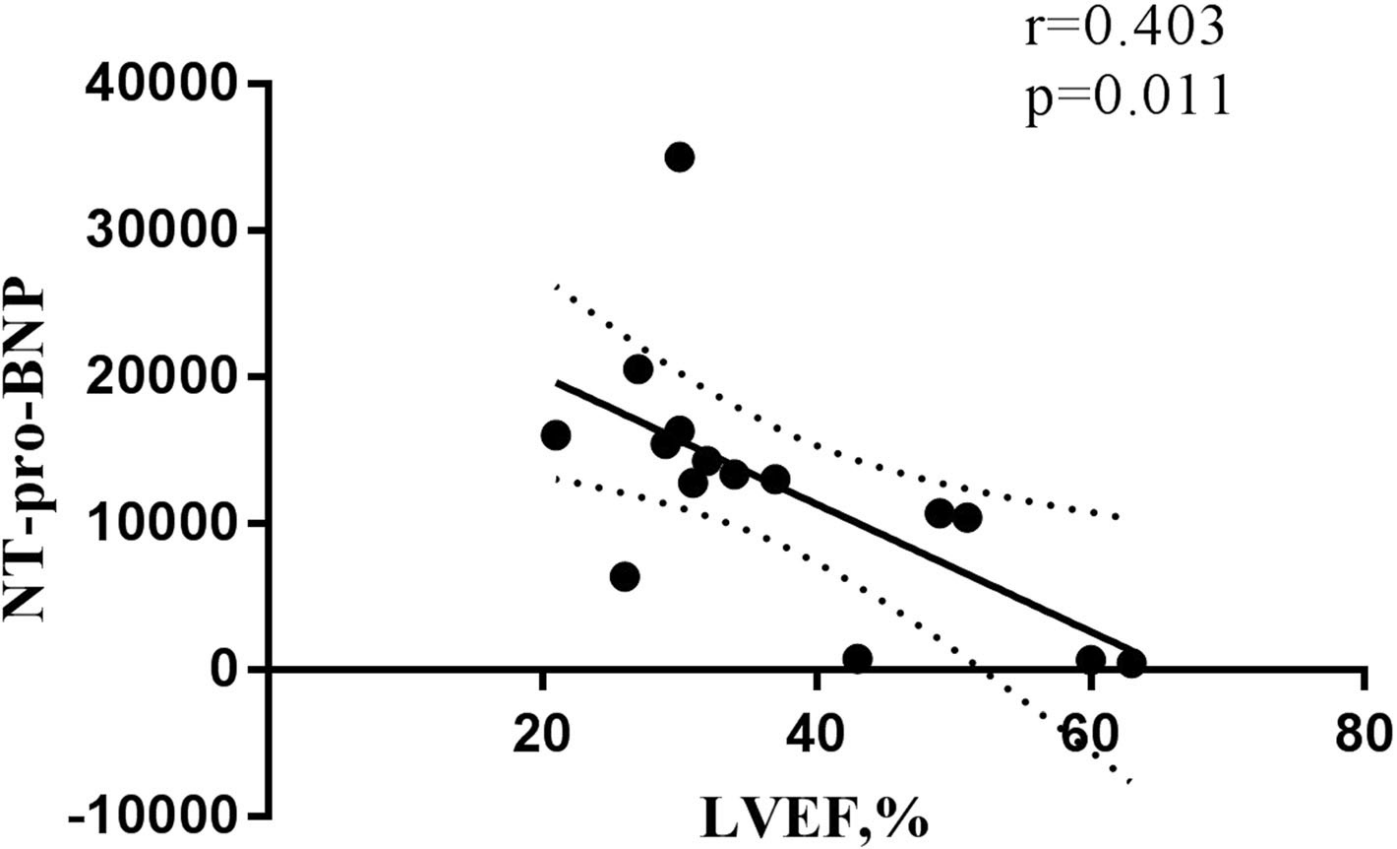
Linear regression showed a negative correlation between LVEF and NT-pro-BNP levels (*r* = 0.403, *p* = 0.011) when NT-pro-BNP levels were above 300 pg/mL

Linear regression showed a negative correlation between LVEF and NT-pro-BNP levels (*r* = 0.570, *p* < 0.0001) in patients with ALCAPA (Fig. 2) and significant correlation when NT-pro-BNP levels were above 300 pg/mL (*r* = 0.403, *p* = 0.011) (Fig. 3). However, no significant correlation was observed between LVEF and NT-pro-BNP (*r* = 0.092, *p* = 0.466) when NT-pro-BNP levels were below 300 pg/mL.

A Spearman correlation test showed no significant correlation between MR grade and NT-pro-BNP levels (*r* = 0.383, *p* = 0.071).

## Discussion

ALCAPA is a rare but potentially fatal congenital coronary malformation associated with early infant mortality and adult sudden death [6]. The degree of coronary collateral circulation determines the type of ALCAPA. In infantile type, the left coronary artery (LCA) flow decreases and may reverse when pulmonary arterial pressure decreases. This leads to myocardial ischemia and infarction in the first or second month of life. Adult type patients have relatively well-established coronary collateral circulation, and this extensive collateral circulation makes it possible to survive beyond infancy [15, 16].

The assessment of collateral circulation development is challenging. Communication and flow from the LCA into the PA can be demonstrated by SSFP cine MR imaging, and the flow can be assessed by fast cine phase-contrast imaging qualitatively and quantitatively. Delayed gadolinium enhancement may help determine myocardial viability. However, MR imaging has significant disadvantages including low spatial resolution and long examination times [17]. Previous studies have indicated that it is reasonable to use 300 pg/mL as cutoff value to “rule out” HF [14]. When interpreting NT-pro-BNP values in ALCAPA patients, the NT-pro-BNP cutoff point (300 pg/mL) may provide quick information about the severity of myocardial ischemia and LV dysfunction. In our study, we have shown a statistically significant difference in LVEF (*p* < 0.0001) between groups with NT-pro-BNP levels above or below 300 pg/mL, and a negative correlation between LVEF and NT-pro-BNP levels (*r* = 0.570, *p* < 0.0001), also in the group with NT-pro-BNP levels above 300 pg/mL (*r* = 0.403, *p* = 0.011). These data indicate that left ventricular dysfunction is more severe in patients with NT-pro-BNP levels above 300 pg/mL. Considering the convenience of measuring plasma NT-pro-BNP levels, NT-pro-BNP may serve as a useful diagnostic biomarker to determine the type of ALCAPA, especially in the non-acute setting when MR imaging is not immediately available.

Patients who developed extensive coronary collaterals could stay asymptomatic before diagnosed with ALCAPA by physical examination in adolescence or adulthood, with little subclinical myocardial ischemia and lower NT-pro-BNP levels [15]. Those who were diagnosed with ALCAPA in infancy or childhood are mostly symptomatic and have higher NT-pro-BNP levels. Considering the difference of diagnosed time of adult and infantile types, it is reasonable that age showed statistical significance in a comparison of the two groups with the cutoff point of 300 pg/mL in our study.

When NT-pro-BNP levels were above 300 pg/mL, a statistically significant negative correlation was observed between age and NT-pro-BNP, indicating that severer subclinical myocardial ischemia and worse heart dysfunction in children and infant who were diagnosed in younger age. No statistically significant correlation between age and NT-pro-BNP was observed when NT-pro-BNP levels were below 300 pg/mL in our study, indicating relatively milder persistent subclinical myocardial ischemia in elder patients due to the extensive coronary collaterals.

In pediatric patients, we should take variability associated with both physiology and methodology into consideration in order to use NT-pro-BNP as a clinical biomarker effectively. In healthy populations, NT-pro-BNP is very high in the first 4 days of life, and then decrease quickly through the first week, followed by a slow progressive decline for up to the first month of life [18]. No significant differences between age and gender were observed in NT-pro-BNP levels after the first month of life [19]. Therefore, when interpreting NT-pro-BNP levels in neonatal ALCAPA patients, the difference in age and methods should be taken into consideration.

MR has been commonly observed in ALCAPA patients. In our study, no significant correlation was observed between MR grade and NT-pro-BNP level. Considering the limitated sample size, further studies should be performed to asses a possible correlation between them.

## Conclusion

Age and LVEF negatively correlate with high levels of NT-pro-BNP in ALCAPA patients and no correlation with age and LVEF in patients with NT-pro-BNP < 300 pg/mL. Further studies are needed to determine whether there is a correlation between MR grade and NT-pro-BNP levels. A possible correlation between MR grade and NT-pro-BNP levels needs more detailed assesment. Larger prospective studies are needed to further assess the value of NT-pro-BNP in ALCAPA patients.

## Data Availability

The datasets used are available from the corresponding author on reasonable request.

## Abbreviations

ALCAPA: Anomalous left coronary artery from the pulmonary artery
BNP: Brain natriuretic peptide
CHD: Congenital heart defects
HF: Heart failure
LCA: Left coronary artery
LVEF: Left ventricular ejection fractions
MR: Mitral regurgitation
NT-pro-BNP: N-terminal pro-brain natriuretic peptide
PA: Pulmonary artery
RCA: Right coronary artery
